# The role of point-of-Care Musculoskeletal Ultrasound for Routine Joint evaluation and management in the Hemophilia Clinic - A Real World Experience

**DOI:** 10.1186/s12891-022-06042-w

**Published:** 2022-12-21

**Authors:** N Gallastegui, BUK Steiner, P Aguero, C Bailey, R Kruse-Jarres, DV Quon, C Hanacek, LM Volland, RFW Barnes, A von Drygalski

**Affiliations:** 1 Department of Medicine, Division of Hematology/Oncology, University of California San Diego, CA San Diego, USA; 2 Department of Medicine, Division of Hematology, The Ohio State University, OH Columbus, USA; 3 Washington Center for Bleeding Disorders, WA Seattle, USA; 4 The Orthopaedic Hemophilia Treatment Center, Orthopaedic Institute for Children, Los Angeles California, USA; 5 Department of General Medical Education, KPC Health. Hemet, CA, USA 1810 Cannon Drive, Suite 1150E, OH Columbus, USA; 6 National Hemophilia Foundation, NYC NY, USA

**Keywords:** Hemophilia, Arthropathy, Hemarthrosis, Joint disease, HJHS, Ultrasound, JADE, MSKUS

## Abstract

**Background:**

The use of musculoskeletal ultrasound (MSKUS) for point-of-care (POC) evaluation of hemophilic arthropathy is growing rapidly. However, the extent to which MSKUS influences clinical treatment decisions is unknown.

**Methods:**

We conducted a three-year, prospective, multi-center study at three hemophilia treatment centers in the United States to evaluate the utilization of POC-MSKUS for routine clinical decision-making in adult persons with hemophilic arthropathy. Bilateral elbows, knees and ankles were assessed clinically [Hemophilia Joint Health Score (HJHS)] and with POC-MSKUS by the Joint _Tissue_Activity and Damage Exam (JADE) protocol at baseline and approximately annually for two additional times. Treatment decisions, including physical therapy (PT) and “medical” (joint injections/aspirations, referrals to orthopedics, changes/adjustments of hemostatic plans, and use of oral anti-inflammatory medications) were recorded in relation to POC-MSKUS.

**Results:**

Forty-four persons [median age 37 years (IQR 29, 51)], mostly with severe Hemophilia A on clotting factor prophylaxis, completed 129 visits, yielding 792 joint exams by POC-MSKUS and HJHS [median at baseline 27 (IQR 18, 42)] over a median follow up of 584 days (range: 363 to 1072). Among 157 management decisions, 70% were related to PT plans (n = 110) and 30% were “medical”. Point-of-care MSKUS influenced 47/110 (43%) PT plans, mostly informing treatment of specific arthropathic joints (45/47 plans) in patients with high HJHS. Physical therapy plans influenced by POC-MSKUS directed more manual therapy/therapeutic exercises, while plans based on physical exam were focused more on global exercises and wellness. Treatment decisions were mostly based on the identification of specific musculoskeletal abnormalities visualized by POC-MSKUS. Of note 20/47 (43%) POC-MSKUS plans included de-escalation strategies, thereby reducing exercise intensity, mostly for joint instability and subclinical hemarthroses. Point-of-care MSKUS also informed 68% (32/47) of “medical” decisions, surprisingly mostly for injections/aspirations and referrals to orthopedics, and not for adjustments of hemostatic treatment. Although not formally studied, ultrasound images were used frequently for patient education.

**Conclusion:**

Routine joint evaluations with POC-MSKUS resulted in few changes regarding medical management decisions but had a profound effect on the formulation of PT plans. Based on these findings, new studies are essential to determine the benefit of MSKUS-informed management plans on joint health outcomes.

## Background

Hemophilic arthropathy (HA) is the consequence of joint remodeling resulting from recurrent hemarthroses in persons with hemophilia (PwH), a rare congenital X-linked bleeding disorder characterized by the lack of coagulation factors VIII or IX [1]. Hemophilic arthropathy most commonly affects the large synovial joints (elbows, knees and ankles) and is the main cause of morbidity in this these patients [2]. The pathobiology of HA encompasses chronic deposition of hemosiderin, synovial inflammatory changes, cartilage degradation and, in late stages, joint destruction [3]. The onset of arthropathy largely depends on the number of joint bleeds per year, and the arthropathy may progress despite prophylactic clotting factor replacement strategies. Therefore, long-term preservation of joint health is an unmet need [4].

Hemophilic arthropathy affects the quality of life of PwH [5, 6]. Current therapeutic efforts are focused on the prevention of HA and further joint deterioration once HA has developed [7, 8]. Current hemostatic strategies are centered on replacement therapy with factor and non-factor products, as well as encouragement of adherence to treatment [9]. Other important interventions beneficial for improving HA and related symptoms are physical therapy [10–13], the use of anti-inflammatory drugs [14] and surgical interventions such as joint replacement [15, 16].

For timely and meaningful management of HA, it is critical to assess the extent and progression of HA regularly. Scores based on clinical joint examination, such as the Hemophilia Joint Health Score (HJHS) [17, 18], are widely used to quantify and evaluate HA. Also, radiographic and magnetic resonance imaging (MRI) scores have been introduced based on visualization of tissue abnormalities associated with HA [19]. Magnetic resonance imaging is currently considered the gold standard [20] for joint evaluation as it can detect early stages of arthropathy [19, 21] and even predict joints at risk for bleeding [22]. However, MRI has a number of drawbacks, such as cost, time required to complete imaging, and the need for sedation in some patients, especially children [1]. While objective imaging is highly desirable to document elements of arthropathy, neither radiographic nor MR imaging are available at the point-of-care (POC). Therefore, musculoskeletal ultrasound (MSKUS) is growing rapidly as a powerful tool for the evaluation of hemarthrosis and painful joint episodes [23–25]. Musculoskeletal ultrasound is also being used more and more for longitudinal joint assessments to document soft tissue proliferation and osteochondral abnormalities [4]. Musculoskeletal ultrasound has also been shown to correlate with MRI [26], even being superior in certain aspects, such as detection of intra-articular blood [27]. Several [2, 28–34] MSKUS protocols (at different levels of validation) have been proposed using scoring algorithms to determine the extent of joint deterioration.

The Joint_tissue_ Activity and Damage Exam (JADE) is a musculoskeletal ultrasound protocol that incorporates direct measurements of tissue abnormalities, such as soft tissue thickness, osteochondral alterations, and cartilage thickness, using specific views [29]. The JADE protocol has been validated by Outcomes Measures in Rheumatology (OMERACT) guidelines [35] on the tissue discrimination level [35], and has demonstrated high intra/inter-rater and inter-operator reliability [29, 36, 37]. Also, the direct tissue measurements correlate well with clinical and functional parameters used to evaluate PwH [38].

A recent international survey found that Hemophilia Treatment Centers (HTCs) use MSKUS increasingly to assist with the management of HA. Most HTCs found MSKUS most useful to rapidly detect hemarthrosis and/or synovitis [4, 39]. Interestingly, POC-MSKUS was used less for longitudinal joint health assessments, perhaps because little is known regarding its potential usefulness for treatment formulations and the impact on outcomes [4, 39]. Here we describe the contributions of POC-MSKUS to patient management ascertained during a prospective study conducted at 3 HTCs, incorporating POC-MSKUS using the JADE protocol for joint examinations into routine comprehensive care.

## Methods

### Patient selection

Patients with hemophilia A or B (factor VIII or IX ≤ 50% of normal) and age ≥ 18 years, were recruited prospectively at three HTCs in the United States of America (Washington Center for Bleeding Disorders, University of California San Diego, Orthopaedic Institute for Children) between May 2016 and April 2019. No patients were excluded, but patients had to have documentation of at least one arthropathic joint suggested by a Pettersson score [40] of ≥ 1 or an HJHS [17] of ≥ 3 based on published correlations between the two joint outcome measures [41, 42]. Patient demographic information, age, type and severity of hemophilia were collected. Patients also were asked to self-report subjectively arthropathic “problem joints”. Patients were followed prospectively for 36 months and subjected to joint health assessments at baseline and approximately annually thereafter. These joint health assessments comprised a pain assessment by Visual Analogue Scale (VAS), HJHS [17], and joint examination with MSKUS/Power Doppler (PD) of both elbows, knees and ankles using the JADE protocol [29].

At the end of each visit, management plans were recorded for each patient capturing the following categories: Joint injection/aspiration, clotting factor and non-factor hemostatic therapy (product, dose and frequency), oral anti-inflammatory medication therapy (product, dose and frequency), physical therapy (frequency and physical therapy modalities) and referrals to other medical or surgical specialties. Non-scripted comments by providers were also collected. After completion of the visit, the medical team had to document if and how joint examination with MSKUS influenced treatment plans.

All subjects signed an informed consent, and the study was approved by the ethics committees and/or institutional review boards of all 3 institutions (Institutional Review Board #:120,510).

### Joint Health evaluation

#### Hemophilia Joint Health Scores

Clinical joint examination was accomplished by performing the HJHS (version 2.1) during every visit for all 6 joints (elbows, knees, and ankles) [17] The HJHS version 2.1 is an established outcome measure, providing a clinical score for each joint summarizing swelling, duration of swelling, pain, strength, loss of range of motion (ROM), muscle atrophy, and crepitus (HJHS per joint: 0 best, 20 worst; total HJHS for 6 joints combined: 0 best, 120 worst). Hemophilia joint health scores were performed by a licensed physical therapist with > 5 years of general practice experience and approximately 2 years of experience with hemophilia patients (BUKS, LMV, CB). The physical therapists were trained in the HJHS acquisition according to instructions and guidance provided by online training and video modules developed by the International Prophylaxis Study Group (http://www.ipsg.ca/publication/hemophilia-joint-health-score-instructional-video-and-manual).

### Musculoskeletal Imaging

Joint examinations with MSKUS were completed during every visit for the same 6 joints using the JADE protocol [29]. All 3 institutions utilized a GE Logiq S8 ultrasound machine (General Electrics, Fairfield, Connecticut) with real-time spatial compound imaging, speckle reduction capabilities, and an 8–15 MHz high-frequency linear transducer using grayscale (B-mode) with PD settings applied in accordance with the manufacturer’s recommendations. Musculoskeletal ultrasound was performed by the physical therapists (BUKS, PA, CB LMV), who had been trained in the CME-accredited course “Musculoskeletal Ultrasound in Hemophilia” at UCSD (https://cme.ucsd.edu/httc/index.html), with a minimum of 3 years of imaging experience. Three providers (BUKS, PA and AvD) are certified in MSKUS through the Alliance for Physician Certification & Advancement, a service of the American Registry for Diagnostic Medical Sonography. Images were discussed with the hematologists (AvD, RK-J, DVQ), trained in the same course, during each visit using the descriptive sections of the JADE protocol (e.g. echogenicity, compressibility of joint space content) as a discussion guide, but also allowing additional comments based on patients’ pathology in structures visible through the JADE views (e.g. tendon strain). A description of the JADE protocol including transducer positions and measurements (soft tissue, cartilage and osteochondral) has been published [29].

### Statistical analyses

Results are expressed as median values with either inter-quartile ranges (IQR) or the range between minimum and maximum values. Groups that were compared were not independent of each other because a patient might occur in one group at one time and in another group at a later evaluation. Therefore, no statistical tests were applied.

We used ranking by percentiles of HJHS scores by joint (ankles, knees, and elbows) at the three different study periods (baseline, midpoint and final) to determine the median percentile of HJHSs for joints that received a joint injection/aspiration.

We used the Spearman rank correlation test to evaluate the association between self-reported arthropathic joints and the study inclusion criteria.

To compare patient characteristics between the three centers at study entry, we used the Kruskal-Wallis test for continuous variables, and the Fisher Exact test for categorical variables.

## Results

### Persons with hemophilia, joint and visit characteristics

Forty-four PwH were enrolled, and 42 completed all 3 visits (baseline and 2 consecutive follow-up visits), accounting for 129 visits with 792 joint exams by POC-MSKUS and HJHS. The median follow-up for each patient was 584 days (range: 363 to 1072), with a median interval between visits of 273 days (range: 106 to 679). Persons with hemophilia and joint characteristics are shown in Table 1. Participants were evenly distributed in between the 3 HTCs (*n* = 15, 15, 14). There were no statistically significant differences at baseline in age, HJHS scores, race/ethnicity, or hemophilia type or severity between the subjects recruited in between centers. The median number of arthropathic joints as per study inclusion criteria was 3 (range: 1 to 6). The median number of self-reported arthropathic joints per patient was also 3 (range: 0 to 8); the median total HJHS was 27 (IQR: 19, 41). There was a strong correlation between the number of self-reported arthropathic joints and the number of arthropathic joints by study criteria per patient at study entry (*r*_s_ = 0.703, *n* = 44, *p* < 0.001), supporting the clinical relevance of HJHS assessments. 66% of the PwH had a history of at least one previous surgical intervention (median = 1; range 1 to 4) involving shoulders, elbows, hips, knees, and/or ankles (Table 2). Altogether, these characteristics are consistent with a broad and heterogeneous spectrum of hemophilic manifestations, representative of a “real world” cohort of adult PwH.

**Table 1:** Baseline patient and treatment characteristics

Number of patients	44
**Demographics:**	
**Age (years) [Median; IQR]**	37 (29, 51)
**Sex [n, %]**	
Male	43 (98)
Female	0 (0)
Transgender	1 (2)
**Race/Ethnicity [n, %]**	
White	26 (59)
Black	4 (9)
Asian	2 (5)
Hispanic	7 (16)
Other	5 (11)
**Hemophilia [n, %]**	
A	35 (80)
B	9 (20)
**Hemophilia severity [n, %]**	
Mild	3 (7)
Moderate	4 (9)
Severe	37 (84)
**Treatment:**	
**Hemophilia Primary Therapy [n, %]**	
**Clotting factor-based therapy**	40 (90)
Prophylaxis	38 (95)
For ≥ 6 months	34 (89)
For < 6 months	3 (11)
On demand	2 (5)
**Gene therapy**	2 (4.5)
**Emicizumab**	2 (4.5)
**Pain/Anti-inflammatory medications [n, %]**	18 (41)
Anti-inflammatory medications	5 (28)
Acetaminophen	2 (11)
Opioids	8 (44)
Anti-inflammatory medications and opioids	2 (11)
Other	1 (6)

**Table 2:** Baseline joint characteristics

**Joint characteristics [Median; Range; IQR; %]**	
**Number of self-identified arthropathic joints**	3 (0, 8)
**Number of arthropathic joints by study definition (Joint HJHS ≥ 3 or Pettersson score ≥ 1)**	3 (1, 6)
**Total HJHS**	27 (19, 41)
**Procedures**	
**Joint procedures, patient**	1 (0, 4)
**Joint procedures, joint**	52
Ankles	14 (27)
Knees	24 (46)
Elbows	7 (13)
Hips / Shoulders	7 (13)
**Joint procedures, type**	
Synovectomy	10 (19)
Fusions	5 (10)
Replacement	24 (46)
Other	13 (25)

**Fig. 1:**
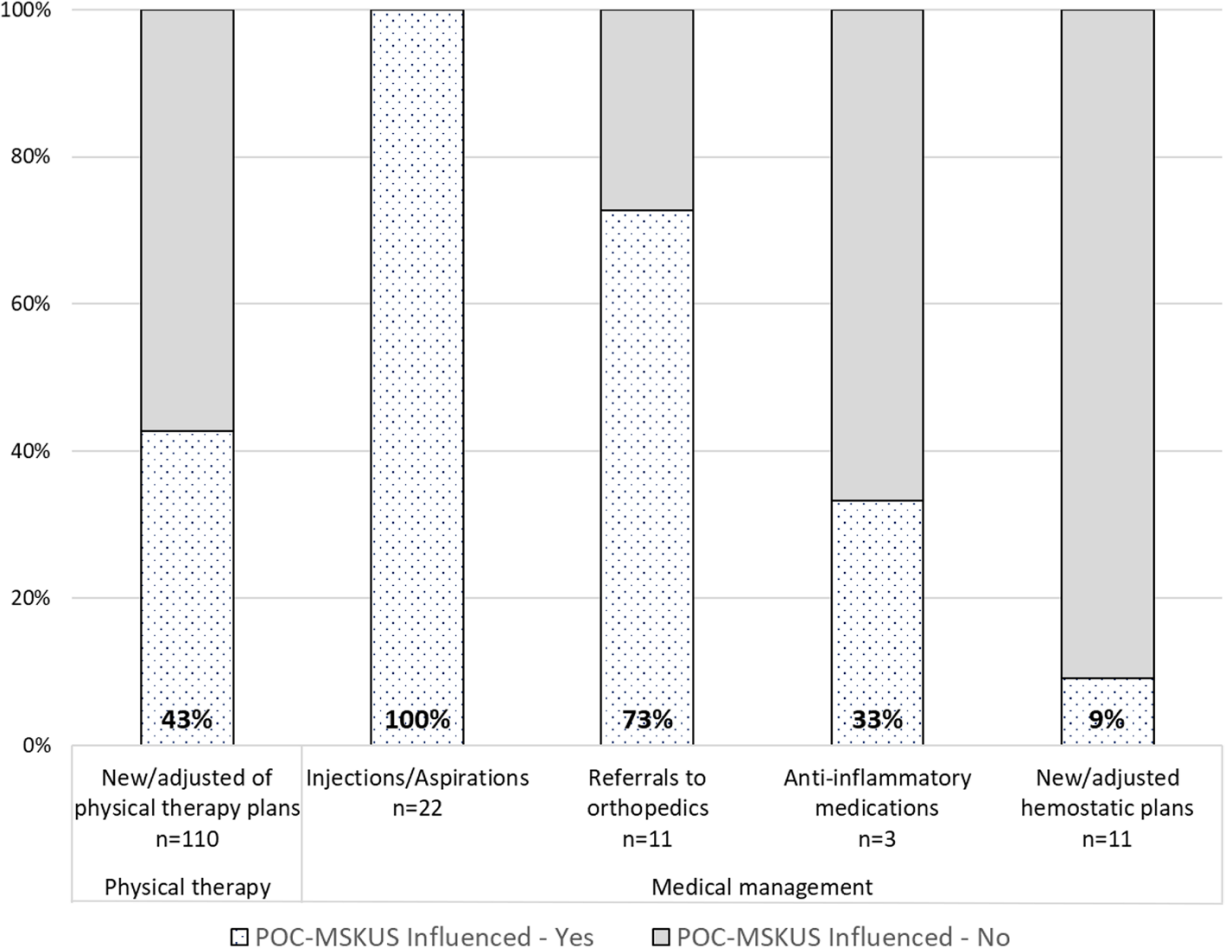
Proportion of management plan decisions informed by Point-of-Care Musculoskeletal Ultrasound throughout the study period

A total of 157 management decisions were formulated in 40/44 PwH: 44 at baseline, 53 at first follow-up, and 60 at second follow-up (study exit). These decisions included new or adjusted physical therapy plans, joint injections/aspirations, referrals to orthopedics, changes/adjustments of hemostatic plans (clotting factor and non-factor-based treatments), and the use of oral anti-inflammatory medications. Point-of-care MSKUS informed 50% (79/157) of management decisions (Fig. 1).

“Medical” management plans (joint injections/aspirations, referrals to orthopedics, changes/adjustments of hemostatic plans, and use of oral anti-inflammatory medications):

### Joint injections/aspirations

Twenty-two injections/aspirations were recommended for 9 PwH during the study period (median of 2 proposed injections/aspirations per patient [range: 1 to 5] and 2 [range 1 to 2] per visit). Most procedures were recommended for ankles (54%), followed by elbows (23%), knees (18%), and shoulders (5%). The procedural indications were made on clinical grounds only (not based on POC-MSKUS findings). Based on patient preference, only 55% (12/22) of the recommended injections/aspirations were performed; all under ultrasound guidance, informing the procedural approach. In addition, the use of POC-MSKUS identified one additional effusion, which was suspicious for subacute bleeding (complex appearance), confirmed by aspiration.

To evaluate the extent of clinical arthropathy of the joints proposed for injection/aspiration, the joints were ranked by HJHS percentile. It appeared that many joints were above the 80th percentile, indicating relatively far advanced arthropathy. In more detail, the HJHSs of elbows, knees and ankles fell on the 86th, 94th, 77th percentile at baseline; (no events), 83th, 68th at the first, and 61th, 86th, 87th at the second follow-up visit, respectively.

### Hemostatic treatment plans, anti-inflammatory medications, and referrals

Only 11 hemostatic treatment plans were adjusted or changed in 9 PwH during the study period. Decisions were based predominantly on clinical assessment (e.g. number of patient-perceived bleeding events) rather than findings with POC-MSKUS. The most common reason for the adjustments during visits was a switch from conventional half-life to extended half-life clotting factor formulations [45% (5/11)], followed by the transition to emicizumab (Hemlibra®, Genentech, South San Francisco, USA) [27% (3/11)], and changes in dose [18% (2/11)] and/or frequency [11% (1/11)] of conventional half-life formulations. There was only one case where an increase in clotting factor dosing was proposed based on POC-MSKUS findings, describing “rapid joint deterioration.”

Only 3 new prescriptions for oral anti-inflammatory medications were provided, whereby POC-MSKUS guided only one decision (strong PD signal).

Seven PwH were referred to Orthopedic Surgery during the study period for evaluation of 11 painful joints (5 knees, 1 elbow, 4 ankles, and 1 hip). Point-of-care MSKUS triggered the referral for 8/11 joint evaluations (73%), documenting pronounced soft tissue proliferation in 6/8 joints (as the main cause for the referral in addition to pain), and severe osteochondral alterations in the remaining 2 joints. Of note, more than half of the joints (5/8) evaluated by orthopedics based on a referral triggered by POC-MSKUS examination proceeded to surgical interventions.

### Physical therapy

A total of 110 PT plans (either new or adjusted from previous) were proposed for 84% (37/44) PwH, occurring during 57% of visits (73/129). The median number of new or adjusted PT plans per patient was 3 (range: 1 to 7) during the study period, with a median of 1 plan per patient for each visit (range: 1 to 3). Overall, POC-MSKUS informed the design of 43% (47/110) PT plans, affecting 60% (22/37) of the PwH at least once (median number of visits = 1, range: 1 to 3).

### Joint-specific vs. global physical therapy plans

**Fig. 2:**
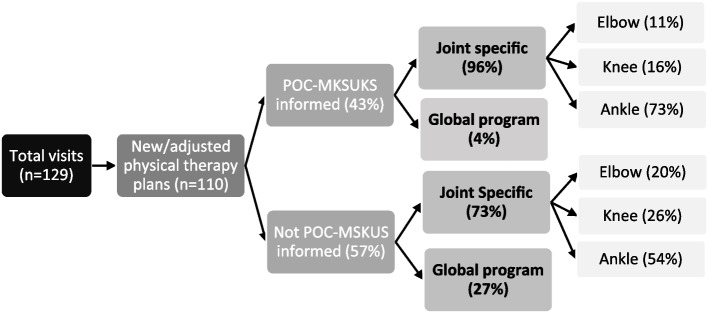
Delineation of physical therapy plans informed by physical exam or Point-of-Care Musculoskeletal Ultrasound (POC-MSKUS).

Most PT prescriptions [83%, (91/110)] were directed to a specific joint, while 17% prescriptions (19/110) encompassed a global wellness/strengthening PT program. When prescriptions were divided into those informed by POC-MSKUS (43%) and those not (57%), targeted joint-specific plans were almost all informed by POC-MSKUS, whereas global PT programs were to a much less extent (96% vs. 4%) (Fig. 2). Notably, the joint that received POC-MSKUS informed PT most frequently was the ankle (Fig. 2).

Also of note, most joint-specific plans [86% (78/91)] were directed to one of the self-reported arthropathic joints, whether or not POC-MSKUS was used to inform plans (POC-MSKUS 80% vs. non POC-MSKUS 95%).

### Physical therapy plans in relation to HJHSs

The median total HJHS for the PwH was 34 (IQR: 28, 48) and 30 (IQR: 19, 38) during visits triggering PT plans informed and uninformed by POC-MSKUS. In contrast, the median HJHS was 20.5 (IQR: 14, 36) in the group that did not receive a PT plan. These findings suggest that joint health status was similarly poor between the groups receiving PT plans whether informed or uninformed by POC-MSKUS, and worse compared to those PwH who did not undergo PT.

Hemophilia joint health scores differed between the groups receiving PT informed or uninformed by POC-MSKUS when the gait component was omitted (34, [IQR: 24, 51] vs. 22 [IQR: 18, 35]). Since the gait score was similar in both groups (median 4, range: 0, 4), this finding suggests that PwH receiving POC-MSKUS informed-PT plans had more pronounced arthropathy compared to those receiving PT plans uninformed by POC-MSKUS.

Overall, the HJHS for PwH who underwent global wellness/strengthening programs (not discriminating between POC-MSKUS informed or not) was higher compared to the group with joint-specific PT plans (38.5, IQR: 32, 46 vs. 30, IQR: 21, 38), suggesting advanced arthropathy status requiring a more functional rehabilitation program.

### Impact of POC-MSKUS on the design of PT plans

It appeared that PT plan compositions were influenced by information derived from POC-MSKUS examinations. Both POC-MSKUS informed (*n* = 47) and -uninformed plans (*n* = 63) were formulated with exercises and PT measures (named “items”) fitting into the following 7 domains: manual therapy, therapeutic exercises, wellness/pain management, balance/gait training, bracing/assistive devices, activities of daily living training and others. Final plans included a single item or a combination of items. The median number of items in POC-MSKUS-informed and uninformed plans were similar [2 (Min = 1, Max = 5)], with a total number of 102 and 123 items, respectively.

**Fig. 3:**
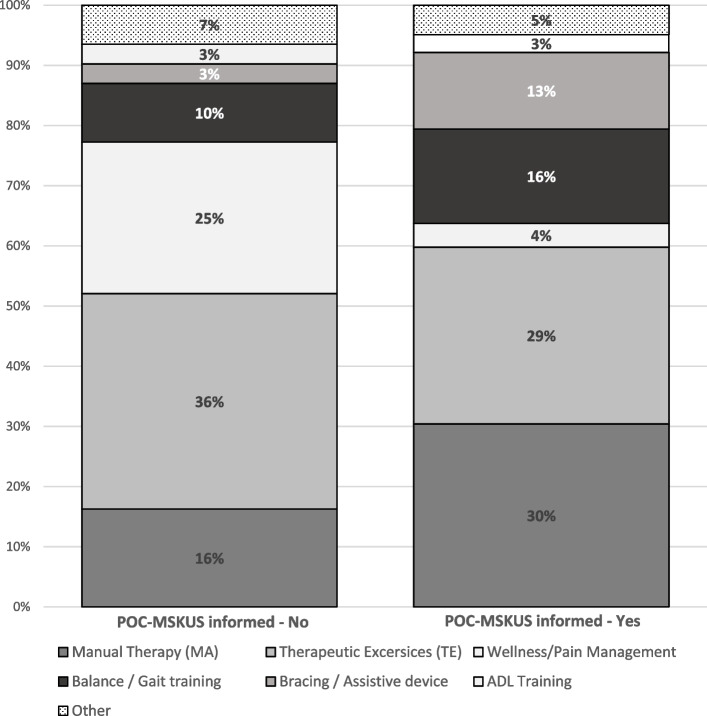
Proportion of physical therapy items in plans informed (*n* = 102) and not informed (*n* = 123) by Point-of-Care Musculoskeletal Ultrasound (POC-MSKUS).

As shown in Fig. 3, plans informed by POC-MSKUS contained a larger proportion of manual therapies (30.4% vs. 15.8%) and bracing/assistive devices (13% vs. 3%) compared to plans uninformed by POC-MSKUS. Plans uninformed by POC-MSKUS had higher proportions of therapeutic exercises (36% vs. 29%) and wellness/pain management measures (25% vs. 13%).

Of interest, the use of POC-MSKUS to devise PT plans (*n* = 47 plans) resulted in considerable de-escalation of therapy plans (45%, 21/47). The most common reason for de-escalation of therapy were findings of severe osteochondral alterations (8/21), followed by “unstable joint” by dynamic arthrokinematics visualized during evaluation (4/21), subclinical hemarthroses (5/21), severe soft tissue inflammation by PD evaluation (1/21) and ‘not specified’ (3/21). In general, specific POC-MSKUS findings described by the physical therapist informed the formulation of 34% (16/47) PT plans. Those findings were listed as severe hemarthritic changes 7/16), the presence of osteophytes (5/16), soft tissue impingements (2/16), tendinopathy (1/16), and ‘unspecified’ (1/16).

### Role of POC-MSKUS for patient education

Evaluation of written provider comments during the course of the study indicated that POC-MSKUS findings prompted educational discussions during 19% (24/129) of all visits, mainly during the 1st and 2nd follow-up visits. Point-of-care MSKUS informed 36 discussions with 18/44 (41%) PwH at least once (median 1, range: 1 to 2). Point-of-care MSKUS informed discussions included education about joint health (*n* = 9), lifestyle modifications (*n* = 10), factor plan treatment adherence (*n* = 9), and rationale for a specific exercise plan (*n* = 8).

## Discussion

This study describes the “practical” use of MSKUS for the management of hemophilic arthropathy, a new modality met with increasing enthusiasm from hemophilia care providers. The need for personalized therapies based on MSKUS findings has been suggested in recent publications based on the ability of MSKUS to detect early pathology [43, 44]. However, insights regarding current practice implementation patterns are largely missing. The most important and surprising finding from this study was that POC-MSKUS applied to routine hemophilia care was used predominantly to inform PT plans during routine comprehensive care visits, rather than medical management plans, such as (non-)factor prophylaxis plans, or oral or local pain management.

Interestingly, the vast majority of PwH (84%) were involved in PT throughout the study period and MSKUS was utilized actively by the physical therapists to devise or adjust plans in a ‘real time’ fashion. Nearly half of the plans were informed by POC-MSKUS, based on direct visualization of joint tissue abnormalities and their integration with clinical findings.

Musculoskeletal ultrasound examinations were performed with the JADE protocol [29], which uses direct measurements for cartilage and soft tissue (synovial) thickness as well as osteochondral alterations, but also offers the opportunity for qualitative assessments and descriptions of findings (for instance content description of joint space-occupying structures such as fluid/bleeds, presence of inflammation by PD signal, or presence of other abnormalities such as tendinopathy or osteophytes). Here, physical therapists based their decisions on the identification of such abnormalities, rather than tissue measurements.

Tissue measurements may be more helpful to quantify the effects of treatment interventions over time. The identification of musculoskeletal abnormalities is thought to contribute to discomfort, or musculoskeletal ailments and should drive the formulation of timely PT decisions. Musculoskeletal ultrasound appeared most useful in informing joint-specific rehabilitation plans for joints with high HJHSs (i.e., more pronounced arthropathy) rather than to devise general wellness measures. Musculoskeletal ultrasound allowed shaping the design of PT plans in targeted fashion with manual therapies, fitting bracing/assisting devices, and gait/balance training.

It is noteworthy that approximately half of the PT plans informed by MSKUS included a de-escalation of the therapeutic intensity previously felt to be safe and appropriate. In this context, subclinical hemarthroses were one of several prominent findings leading to less aggressive joint rehabilitation. Also, plans informed by POC-MSKUS were more specific addressing particular findings, prescribing directed therapies such as manual therapy, bracing/assistive devices, and gait/balance training exercises. On the other hand, PT plans uninformed by POC-MSKUS provided a more general approach to exercise, wellness and pain mitigation. In this context it is important to state that PT interventions in hemophilia are highly effective [10] and that MSKUS examinations in conjunction with functional/physical assessments are very useful to address synovitis [45].

There is mounting evidence that ankles are now superseding the knee as the arthropathic joint indicator for PwH [46], and our study corroborated this. Ankles were most affected and treated with PT, followed by knees and elbows. Of interest, we reported previously that the number of joints without measurable cartilage in ankles was much higher compared to elbows and knees when applying JADE measurements to the same cohort of PwH [38].

The lack of influence of POC-MSKUS on medical decision-making, especially regarding clotting factor adjustments, was notable and may be because the study cohort only included adult subjects, many with established arthropathy. It is conceivable that the benefit of adjusting hemostatic management, especially in subjects with advanced arthropathy, was perceived as less impactful than PT offerings to strengthen musculoskeletal fitness and overall physical well-being. The use of MSKUS in children or adolescents with relatively preserved joints may influence management decisions differently where early findings of osteochondral damage or synovial swelling may trigger the more aggressive use of clotting factor replacement, rather than PT alone. However, it must be emphasized that there are no studies to date showing that MSKUS-guided management improves joint outcomes. The indication for corticosteroid joint injections was almost always clinical, although POC-MSKUS was adopted for needle guidance in all instances, probably based on the growing knowledge that accuracy of needle placement results in improved pain relief, with very good success in hemophilic joints [47].

This study revealed several important aspects related to the potential of MSKUS in routine hemophilia care. First, physical therapists were the predominant specialty to embrace decision-making based on POC imaging findings, using it to inform management plans. These findings align with results from an international survey of HTCs by the International Prophylaxis Study group, identifying physical therapists as the clinical staff performing MSKUS > 50% of the time in routine practice [39]. Second, direct visualization of joint images and pathology may not only help physical therapists but, if shared with patients, may be a very useful tool to generate “buy-in” for treatment decisions and improve adherence to treatment plans. In our study, POC-MSKUS was perceived as a valuable tool for patient engagement and education. Sharing joint visuals enabled informed discussions surrounding joint health and rationales for lifestyle modifications, specific treatment decisions, and adherence during ~ 20% of visits. While this has been discussed among experts, no formal study has been performed to show the value of MSKUS-guided patient education on treatment adherence. Based on the high proportion of educational discussions triggered by MSKUS findings reported here, we propose future studies to document benefits of MSKUS-guided discussions as is already being pursued in patients with rheumatoid arthritis [48–50]. The application of MSKUS may enhance the armamentarium for successful counseling, improving patient compliance with proposed hemophilic management strategies [51]. Third, the fact that MSKUS was used so frequently to guide (especially PT) management decisions in routine care, highlights the need for studies to assess the effects of MSKUS-guided management on long-term joint outcomes.

This study has several limitations. First, the results only apply to adult PwH with more advanced hemophilic arthropathy and not to children or younger populations with hemophilia and lesser arthropathy. Second, the evaluation of study results is often more descriptive than quantitative, based on the unanticipated finding that MSKUS was used disproportionally for the formulation of PT plans involving the dissection of written notes. Third, MSKUS and joint examinations were both performed by the physical therapists in all centers, and not always in the same sequence, in alignment with unpredictable flow during comprehensive care clinic visits. Therefore, one cannot exclude that management approaches were biased by knowledge derived from both exam modalities.

## Conclusion

This study found that MSKUS, when incorporated into comprehensive hemophilia care during routine follow-up visits, was utilized most frequently to inform joint-specific PT plans, rather than leading to adjustments in hemostatic support. We expect that the continued amalgamation of imaging findings using POC-MSKUS and clinical findings (such as HJHS) will lead to improvements in hemophilia management, especially in the area of PT. These findings should stimulate future studies designed to identify the benefit of MSKUS-informed management plans on joint health outcomes.

## Data Availability

The datasets used and/or analyzed during this current study are available from the corresponding author on reasonable request.

## Abbreviations

MUSKUS: Musculoskeletal ultrasound
POC: Point-of-care
HJHS: Hemophilia Joint Health Score
JADE: Joint _Tissue_Activity and Damage Exam
PT: Physical therapy
IQR: Interquartile
PwH: Person with hemophilia
HA: Hemophilic arthropathy
MRI: Magnetic resonance imaging
OMERACT: Outcomes Measures in Rheumatology
HTC: Hemophilia Treatment Center
VAS: Visual Analogue Scale
ROM: Range of motion
PD: Power doppler
